# Evaluation of the effectiveness of manual chest physiotherapy techniques on quality of life at six months post exacerbation of COPD (MATREX): a randomised controlled equivalence trial

**DOI:** 10.1186/1471-2466-12-33

**Published:** 2012-07-02

**Authors:** Jane L Cross, Frances Elender, Gary Barton, Allan Clark, Lee Shepstone, Annie Blyth, Max O Bachmann, Ian Harvey

**Affiliations:** 1 University of East Anglia, Norwich, UK; 2 School of Allied Health Professionals, University of East Anglia, Queens Building, Norwich, Norfolk, NR4 7TJ, UK

## Abstract

**Background:**

Manual chest physiotherapy (MCP) techniques involving chest percussion, vibration, and shaking have long been used in the treatment of respiratory conditions. However, methodological limitations in existing research have led to a state of clinical equipoise with respect to this treatment. Thus, for patients hospitalised with an exacerbation of Chronic Obstructive Pulmonary Disease (COPD), clinical preference tends to dictate whether MCP is given to assist with sputum clearance. We standardised the delivery of MCP and assessed its effectiveness on disease-specific quality of life.

**Methods:**

In this randomised, controlled trial powered for equivalence, 526 patients hospitalised with acute COPD exacerbation were enrolled from four centres in the UK. Patients were allocated to receive MCP plus advice on airway clearance or advice on chest clearance alone. The primary outcome was a COPD specific quality of life measure, the Saint Georges Respiratory Questionnaire (SGRQ) at six months post randomisation. Analyses were by intention to treat (ITT). This study was registered, ISRCTN13825248.

**Results:**

All patients were included in the analyses, of which 372 (71%) provided evaluable data for the primary outcome. An effect size of 0·3 standard deviations in SGRQ score was specified as the threshold for superiority. The ITT analyses showed no significant difference in SGRQ for patients who did, or did not receive MCP (95% CI −0·14 to 0·19).

**Conclusions:**

These data do not lend support to the routine use of MCP in the management of acute exacerbation of COPD. However, this does not mean that MCP is of no therapeutic value to COPD patients in specific circumstances.

## Background

Chronic obstructive pulmonary disease (COPD) is characterised by exacerbations some of which result in increased cough and excessive sputum production caused by mucus hyper-secretion and ciliary dysfunction. Manual chest physiotherapy (MCP) involves external manipulation of the thorax using percussion and vibration techniques. Their purpose of these is to intermittently to apply kinetic energy to the chest wall to dislodge bronchial secretions. The patient then clears these secretions with an expiratory manoeuvre such as the forced expiration technique (FET). The assumption underlying the use of MCP is that removing sputum from the airway improves ventilation perfusion ratios and thus lung function. However, reviews of clinical trials report that although airway clearance techniques may improve sputum expectoration, there is no high quality evidence of either short or long term value [1-4].

Methodological limitations inherent in existing studies include; heterogeneous populations, small samples, unstandardised interventions, and confining evaluations to short term outcomes. Thus, there is clinical equipoise about whether MCP confers any benefit to patients with COPD. Consequently, the latest UK guidelines on the management of COPD call for further research on the effectiveness of such physiotherapy techniques [5]. This randomised trial, funded by National Institute of Health Research Health Technology Assessment, addresses the limitations of previous research by standardising the delivery of MCP and obtaining a sample size sufficient size to detect long term clinical effectiveness or equivalence for a patient-orientated, long term outcome.

The full report [6] is available as http://www.hta.ac.uk/1416. This paper summarises the efficacy of MCP administered to patients hospitalised with COPD exacerbation on disease-specific quality of life (QOL) at six months post intervention.

## Methods

### Study design and patients

The MATREX trial was a pragmatic, multicentre, randomised controlled trial powered for equivalence. Between November 21, 2005, and April 30, 2008, 526 patients were enrolled in four centres in the UK. Patients who were admitted to hospital with an exacerbation of COPD were eligible for inclusion in the trial. We excluded patients with any contraindication to the use of MCP techniques* or with no evidence of excess sputum production on auscultation. * Osteoporosis, haemoptysis, bronchial hyper-reactivity, respiratory system malignancy, raised intracranial pressure, uncontrolled hypertension (diastolic > 110), pulmonary embolism, coagulopathy (platelets <50 and/or INR ≥3), bronchopleural fistula, subcutaneous emphysema and left ventricular failure as primary diagnosis Our primary objective was to assess the effect, if any, of MCP administered to patients hospitalised with COPD exacerbation on disease-specific quality of life at six months post randomisation.

Secondary objectives were to describe the components of MCP given to patients hospitalised with COPD including position selection, duration and frequency of treatment, and to describe concurrent changes in oxygen saturation. The protocol was approved by a NHS multi-centre research ethics committee (reference 06/Q0101/140). We obtained written informed consent from all patients.

### Procedures

Before the trial, a MCP treatment protocol was developed with the physiotherapists who would be delivering the intervention at trial sites (full details of the intervention and control are available in [6].). This reflected consensus on best practice regarding the essential elements of MCP and clarified potential areas of ambiguity [3,7]. The protocol’s aim was to clearly define the MCP to be delivered whilst allowing sufficient flexibility to preserve the profession’s ethos of providing treatment according to individual need. By consensus the protocol included the Active Cycle of Breathing Technique (ACBT) [8]. This comprises breathing control, thoracic expansion exercises and FET. A list of potential adverse events and associated symptoms [3] was included, along with recommended actions should any of these occur. The protocol emphasised defining the circumstances under which participants in the control arm would receive MCP, that is, if the physiotherapist or attending physician felt their condition had deteriorated to the extent that MCP was warranted. Essentially, these circumstances constituted a clinical working definition of respiratory failure (see consort diagram).

Adult respiratory ward admission lists at participating hospitals were screened to identify potential study participants. Eligible patients were randomised by telephone using a voice-activated, automated system to stratify by site (block size six). Trial arm allocation was undertaken by an individual not involved in the recruitment process and communicated to participants after their baseline data had been collected. Patients allocated to the intervention arm were guided to perform ACBT whilst the physiotherapist delivered MCP. Sputum volume [9,10] and oxygen saturation [11] are recommended as indicators of physiological impact of MCP. Therefore, oxygen saturation was monitored with a finger pulse oximeter and any sputum produced during treatment was collected. Following MCP, the physiotherapist provided the patient with advice on positioning regarding continuation of ACBT and provided an information sheet summarising this advice. The patient was asked to continue to collect all further expectorant produced during the remainder of their hospital stay. The content, number and duration of further MCP treatments during hospitalisation were at the discretion of the physiotherapist, according to perceived clinical need. For control arm patients the physiotherapist provided guidance on the elements of ACBT and advice on suitable positions to assist with sputum clearance and information sheet summarising this advice. Their oxygen saturation was recorded at baseline only and the patient was asked to collect any expectorant produced during their hospital stay. For six months post-randomisation, patients re-admitted to hospital with an exacerbation of their COPD continued to be treated according to the trial arm to which they had been allocated.

### Outcome measures

The primary outcome measure was change in the Saint Georges Respiratory Questionnaire (SGRQ) score six months after randomisation. The SGRQ is specifically designed for patients with COPD, provides an effective measure of health-related quality of life during acute exacerbations [12] and is a valid predictor of mortality [13-16]. Secondary outcome measures included the Breathlessness Cough and Sputum Scale (BCSS) [17], the EuroQol (EQ-5D) quality of life index [18] and the EQ visual analogue scale (VAS) [19] these measures were recorded during hospitalisation. As some research suggested lung function measures were useful predictors of morbidity but of little value in predicting QOL [20,21] the Medical Research Council (MRC) dyspnoea scale [22] was included as a baseline indicator of severity of disease. We followed up patients six months after enrolment by postal questionnaire to obtain information on COPD-specific QOL and other secondary outcomes. Finally, the number of days spent in hospital during the full six month study period was obtained retrospectively for each trial participant by scrutinising hospital databases at the end of follow up.

### Statistical analysis

Sample size was based on the primary outcome measure, SGRQ score. Treating this study as a nonsuperiority trial, with an effect size of 0·3 standard deviations (typically considered small [23]) as the threshold for superiority then, and assuming a true difference of zero in the population (90% power, 5% significance), a total of 233 subjects in each arm were required. To allow for a 15% drop out rate, we aimed to recruit 275 participants to each study arm, resulting in a total target sample size of 550 participants. Analyses of accumulating data were prepared by the trial statistician and reviewed at least once per year by an independent data monitoring committee.

Baseline comparability between the treatment arms was evaluated by summarising and comparing means and standard deviations (SDs) for continuous variables or numbers and percentages for categorical variables. Analyses were based on intention to treat (ITT). 95% confidence intervals (CI) were estimated for mean difference between the treatment arms. Equality was regarded as a difference in effect size of 0·3 or less in absolute value; that is, if the upper limit of the 95% CI was less than 0·3 and that the lower limit was greater than −0·3. The effect size was defined as the mean difference divided by the pooled, over treatment arm, SD of the outcome. No adjustment for multiple testing was made. Analyses of all but one of the outcome measures were based on an analysis of covariance, with treatment as a fixed effect and baseline scores and site as covariates. Analysis of the number of days in hospital was based on a negative binomial regression model, with treatment as a fixed effect, site as a covariate and no baseline covariate. A pre-planned subgroup analysis of the primary outcome by sputum volume (15mls or less versus more than 15mls) was undertaken by testing for an interaction between the subgroup and the treatment arm in an analysis-of-covariance model, with treatment as a fixed effect and baseline scores, site and subgroup as covariates. All statistical analyses were undertaken using the STATA (Version 9.1 SE) statistical software package (STATACORP LP, Texas, USA).

## Results

**Figure 1:**
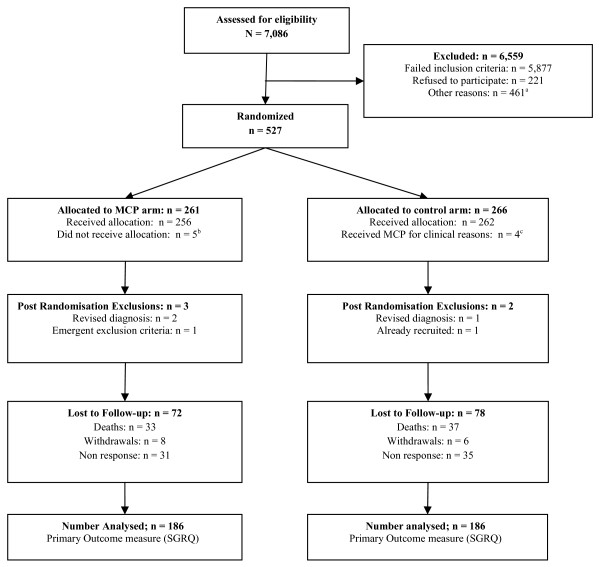
**Trial profile.**^**a**^ Being discharged (n = 241), no physiotherapist available (n = 73), not under care of Respiratory Consultant (n = 55), lives out of area (n = 51), already seen by a physiotherapist (41). ^**b**^ No physiotherapist available (1) patient refused treatment (4). ^**c**^ Clinical working definition of respiratory failure. **ALL** the following criteria were required to switch arm: i) clinical evidence of sputum retention (e.g. auscultation, chest x ray). ii) arterial blood gases: pH less than 7.26. iii) arterial blood gases: rising CO_2._ iv) already receiving controlled oxygen therapy. v) already receiving other supportive treatment(s).

Figure 1 shows the trial profile. The majority of respiratory admissions screened at participating sites were either for patients who did not have COPD, or the reason for their admission was not a COPD exacerbation (85%). The remaining exclusions were due to clinical contra-indications for MCP (8%) or inability to give informed consent (7%). 748 patients were approached to participate in the study, 526 of whom gave their consent (71%). Nine participants did not receive the intervention to which they had been allocated. Four patients randomised to the control arm received MCP for clinical reasons, four patients allocated to receive MCP declined treatment, and one was discharged before the physiotherapist had time to treat them. There were five post randomisation exclusions due to retrospective changes in diagnosis (3), emergent contra-indication to MCP (1), and inadvertent repeat recruitment during subsequent hospitalisation (1). Other losses to follow up comprised death during the six month follow up (70), patient-initiated withdrawal (14), and non-response to questionnaires at six months post-randomisation (66). This equates to a retention figure of 71% for the primary outcome measure at the study end point (372), with similar retention rates for the two arms. Patients’ baseline characteristics were well balanced between treatment groups (Table 1).

**Table 1:** Baseline characteristic of patients enrolled into the MATREX trial

	**MCP arm**			**No MCP arm**		
	**(n = 258)**			**(n = 264)**		
	**N**	**Mean**	**SD**	**N**	**Mean**	**SD**
**Age (years)**	258	69.08	9.85	264	69.58	9.51
**SGRQ symptom score**	249	79.23	14.42	255	79.61	14.18
**SGRQ activity score**	249	84.97	15.46	258	84.10	15.87
**SGRQ impact score**	249	56.58	19.13	258	57.57	18.85
**SGRQ – Total score**	249	68.94	14.66	255	69.13	14.76
**BCSS score**	249	6.23	2.11	256	6.44	2.18
**Oxygen Saturation (%)**	254	92.33	3.67	252	92.77	5.03
**Sputum (mls)**	240	8.17	11.09	255	7.89	9.63
**EQ-VAS**	196	44.95	21.03	202	46.64	21.42
**EQ-5D Score**	199	0.45	0.32	202	0.43	0.36
		**n/N**	**%**		**n/N**	**%**
**Female**		115/258	44.57		109/264	41.29
**Current smoker**		43/221	19.46		49/224	21.88
**Ex smoker**		175/221	79.19		172/224	76.79
**Never smoked**		3/221	1.36		3/224	1.34
**Sputum >15 mls**		38/240	15.83		42/255	16.47
**JPH**		62/258	24.03		65/264	24.62
**NNUH**		77/258	29.84		79/264	29.92
**QEH**		37/258	14.34		36/264	13.64
**UHA**		82/258	31.78		84/264	31.82
**MRC score**						
**1**		0/250	0.00		1/255	0.39
**2**		11/250	4.40		14/255	5.49
**3**		27/250	10.80		27/255	10.59
**4**		68/250	27.20		75/255	29.41
**5**		144/250	57.60		138/255	54.12

### MCP treatment

Over the three year study period, 257 participants received 658 sessions of MCP (Table 2). The numbers of MCP sessions administered to patients ranged between 1 and 25, with the majority receiving 2 or 3 sessions between randomisation and the end of the six month follow up. In the majority of sessions (61%) the physiotherapist selected two different positions in which to place the patient. Whilst the length of time spent performing MCP varied considerably (1–41 minutes), half of all sessions lasted between 11 and 19 minutes. With respect to oxygen saturation, 41% of MCP sessions were associated with decreasing oxygen level although only 6.6% resulted in a change of 4% or more to a value less than 90%, 39% resulted in no change and 19% recorded an increase in oxygen saturation by the end of treatment. This equates to a mean oxygen saturation pretreatment of 92·0%, falling to 91·3% after MCP. Shortness of breath reported by patients and considered as an adverse event was accompanied by varying degrees of reduced oxygen saturation (−18% to 0%). Adverse events comprised increased shortness of breath (5), pain (5), arrhythmia (3), bronchospasm (1), and thoracic haematoma (1).

**Table 2:** Summary of MCP Treatment parameters (N = 658 sessions)

**MCP treatment parameter**	**Min**	**Max**	**Mean/median**	**Breakdown of parameter: N (% total sessions)**			
Number of MCP sessions/patient	1	21	2.53/2	**N. sessions per patient**	**N. patients**	**N. sessions**^a^	**% Total sessions**
					**(total = 257)**	**(total = 658)**	
				1	97	97	14
				2	70	140	21
				3	47	141	22
				4	20	80	12
				5	6	30	5
				6	3	18	3
				7	5	35	5
				8 or more	9	117	18
Number of positions/session	1	3	1.91/2	1 position: 248 sessions (38%)			
				2 positions: 404 sessions (61%)			
				3 positions: 6 sessions (1%)			
Time taken per session	1	41	11.9/11	Less than 5 minutes: 14 sessions (2%)			
				5 − 10 minutes: 266 sessions (40%)			
				11 − 19 minutes: 323 sessions (49%)			
				20 − 25 minutes: 44 sessions (7%)			
				26 or more minutes: 11 sessions (2%)			
O_2_ saturation (%) - immediately prior to MCP	74	100	92.0/93	Less than 85%: 30 (4%)			
				85% to 89%: 111 (17%)			
				90% to 94%: 413 (63%)			
				95% to 100%: 98 (15%)			
O_2_ saturation (%) --lowest during MCP	69	99	91.3/92	Less than 85%: 44 (7%)			
				85% to 89%: 130 (20%)			
				90% to 94%: 385 (58%)			
				95% to 100%: 93 (14%)			
O_2_ saturation (%) - change during MCP	−18	+13	−0.7/0	Drop in O_2_ saturation: 268 (41%)			
				No change in O_2_ saturation: 258 (39%)			
				Increase in O_2_ saturation: 126 (19%)			
Deviations from MCP Treatment Protocol	N = 258			One position only: 248 (38%)			
				O_2_ saturation not recorded: 6 (<1%)			
				Patient declined treatment: 4 (<1%)			
Alternative positions selected	N = 44			Upright: 31 (5%)			
				Leaning forward: 10 (2%)			
				Flat on back: 3 (<1%)			

^a^ Numbers quoted comprise the total number of sessions received by trial participants between 1^st^ December 2005 and 30^th^ October 2008. This includes MCP given at subsequent admissions during the 6 month follow up.

### Effectiveness analyses

No statistically significant differences were found in SGRQ total score, either unadjusted or adjusted for baseline values and hospital site (Table 3). In the unadjusted analysis the mean difference was −0·36 (−4·31 to 3·59) and for the adjusted analysis it was 0·51 (−2·67 to 3·69). These equate to effect sizes of −0·02 (−0·22 to 0·19) and 0·03 (−0·14 to 0·19) respectively. With respect to SGRQ sub-scores, both unadjusted and adjusted CIs are also within the predefined limits of equivalence. Adjusted subscore differences comprised; symptom (0·87, −3·50 to 5·25), activity (−0·36, −3·76 to 3·04) and impact (0·43, −3·29 to 4·14). No statistically significant differences were found (unadjusted or adjusted) in any of the secondary outcome measures (Table 4). Adjusted differences comprised; 0·01 (−0·54 to 0·56) for BCSS, −0·01 (−0·07 to 0·06) for EQ5-D and 2·65 (−2·35 to 7·65) for EQ5-D VAS. The mean number of admissions during the six months following randomisation was 3.89 in the non MCP group and 3.47 in the MCP group. The corresponding number of nights in hospital was 15·95 for the MCP group and 16·98 for the non-MCP group. This equates to an incidence rate ratio (IRR) of 1·07 (0·91 to 1·24). No significant interactions were found in the subgroup analysis of SGRQ by sputum volume (data not shown).

**Table 3:** Primary Outcome measure results

	**MCP arm**			**No MCP arm**			**Unadjusted analysis no MCP versus MCP**			**Adjusted analysis**^**a**^**no MCP versus MCP**		
	N	Mean	SD	N	Mean	SD	Mean difference	95% CI	*p*-value	Mean difference	95% CI	*p*-value
**SGRQ -Total score**	186	63.88	19.05	186	63.52	19.68	−0.36	−4.31,3.59	0.8573	0.51	−2.67,3.69	0.753
**Effect size**							−0.02	−0.22,0.19		0.03	−0.14,0.19	
**SGRQ -Symptom score**	186	68.38	23.13	186	68.40	23.01	0.02	−4.68,4.73	0.9925	0.87	−3.50,5.25	0.695
**Effect size**							0.00	−0.20,0.21		0.04	−0.15,0.23	
**SGRQ -Activity score**	188	82.49	18.81	187	80.91	19.74	−1.58	−5.50,2.34	0.4279	−0.36	−3.76,3.04	0.836
**Effect size**							−0.08	−0.29,0.12		−0.02	−0.20,0.16	
**SGRQ -Impact score**	188	51.53	22.58	187	51.60	22.50	0.07	−4.51,4.65	0.9752	0.43	−3.29,4.14	0.822
**Effect size**							0.00	−0.20,0.2		0.02	−0.15,0.18	

^**a**^ difference adjusted to take into account baseline value and hospital site.

**Table 4:** Secondary Outcome Measures results

	**MCP arm**			**No MCP arm**			**Unadjusted analysis no MCP - MCP**			**Adjusted analysis**^**a**^**no MCP - MCP**		
	N	Mean	SD	N	Mean	SD	Mean difference	95% CI	*p*-value	Mean difference	95% CI	*p*-value
BCSS	175	5.60	2.96	179	5.66	2.84	0.06	−0.55,0.66	0.8577	0.01	−0.54,0.56	0.978
Days in hospital ^b^	258	15.95	16.49	264	16.98	18.04				1.07 ^c^	0.91,1.24	0.4209
EQ-VAS	167	51.29	20.97	173	52.25	19.65	0.96	−3.37,5.29	0.6630	2.65	−2.35,7.65	0.297
EQ-5D Score	209	0.48	0.33	207	0.45	0.35	−0.03	−0.10,0.04	0.3720	−0.01	−0.07,0.06	0.886

^a^ difference adjusted to take into account baseline value and hospital site.

^b^ analysed with a negative binomial regression model.

^c^ Incidence Rate Ratio (IRR).

## Discussion

### Baseline characteristics

This study found SGRQ scores at baseline between five and ten times higher than reported by previous studies in similar settings suggesting our population had a poorer quality of life [24,25]. This perhaps reflects recent improvements in medical treatment with bronchodilators and steroids) that keep people out of hospital for longer until their condition is more severe. Anecdotal evidence suggests there has been an increasing trend for admitted patients to be very sick with end stage disease and multiple co-morbidities. However, the study death rate of 13% is consistent with others reported in the literature [24,26].

### MCP treatment protocol

The MCP treatment protocol was designed both to reflect current practice and to comply with the best available research evidence at the time. Physiotherapists’ high level of adherence indicates that they found this protocol acceptable and so our aim to standardise the study intervention was achieved. With respect to the short term physiological effect of MCP, we found a mean reduction in oxygen saturation of 0·7%.

However there was a wide variation in individual responses, ranging from −18% to 15%. Whilst MCP has been linked to clinically significant falls in oxygen saturation [27] interpretation of our results is difficult because treatment did not occur in isolation. The selection of particular positions and changes to position could also have altered lung ventilation/perfusion ratios. Nonetheless, our findings do suggest that oxygen de-saturation is more common than previously reported, supporting the routine use of oxygen saturation monitoring during MCP, both to identify patients who need oxygen and assess the effect of the MCP itself. The relatively high baseline SGRQ scores found amongst our trial participants indicate a significant level of impairment and there is little robust information to guide clinicians on the risk of significant de-saturation in this patient group.

### Recruitment and retention

The study successfully recruited 526 individuals in from 4 sites in just over 29 months, with the primary outcome recorded for 372 individuals. This was less than our target of 466, hence in order to ensure that we minimised our chance of a type II error we carried out a sensitivity analysis by imputing the incomplete data using multiple chain equations in STATA using all available baseline data in order to base the analyses on all 522 individuals. The results of this were in keeping with the conclusions of the presented analysis. Hence, it is unlikely that the results are due to a type II error.

### The effectiveness of MCP treatment

This study found no gain in long term respiratory quality of life when MCP was included in the physiotherapy management of acute exacerbation of COPD. After adjusting for baseline, the mean difference in SGRQ score at six months was within our pre-specified limits of equivalence. This finding also excludes the minimum clinically important difference (MCID) of four points in SGRQ score [28,29] although it should be noted that the trial was not powered to demonstrate equivalence for this measure. Differences in SGRQ sub-scores also indicate statistical equivalence. Whilst the upper limits of the 95% CI for symptom and impact sub-score did achieve the MCID these differences not statistically significant (*p* = 0·70 and 0·82 respectively). The choice of a quality of life measure as the primary outcome to measure effectiveness is unusual as previous literature has focused on short term physiological measures such as FEV1, oxygen saturation and sputum volumes as measures of efficacy. However short term efficacy may be of little value to the patient unless there is longer term effectiveness. In order to assess this longer term effectiveness QOL is an appropriate patient reported outcome measure. Measure related to short term efficacy such as oxygen saturation and sputum volumes were collected but none of the secondary outcome measures yielded statistically significant results. Although the incident rate ratio for the number of nights in hospital indicates that, on average, the non-MCP group spent 7% longer in hospital, this too was not significant (*p* = 0·42).

### MCP versus ACBT

There is evidence that MCP is used less than before whilst the active cycle remains the treatment of choice. A survey of physiotherapists working in UK acute admitting hospitals [26] asked which physiotherapy treatments they employed to treat COPD exacerbations and with what frequency (n = 146). More than three quarters (77%) responded that they did treat this patient group and that ACBT was employed in the vast majority of cases (88%). A significantly smaller proportion reported using manual techniques “always or often” in conjunction with ACBT (26% vibrations, 8% percussion, 11% shaking) whereas 66% reported using MCP techniques “sometimes or rarely”. In contrast A survey of Canadian therapist found that less than half used manual techniques (42%) relying more extensively on early mobilisation and exercise training [30]. The present study employed ACBT in both trial arms comparing ACBT plus or minus MCP. Thus there remains a need to evaluate the effectiveness of ACBT compared to no ACBT. The high level of adherence to the MCP treatment protocol used in this trial suggests it would be acceptable amongst the profession in usual practice. There is also a need to evaluate the mode of delivery for ACBT. Our results suggest that a short teaching session on ACBT and several sessions of ACBT performed with the support from a physiotherapist have the same effect on QOL after six months. Given recent trends of increasingly severe hospital admissions for COPD, future research regarding physiotherapy interventions with this patient population should focus on examining the effectiveness of ACBT provided in primary care settings.

## Conclusions

### Implications for healthcare

The National Strategy for COPD in England was developed by the Department of Health and went to consultation early in 2010. This was the first national strategy for a respiratory disease in England. One of its defined remits is to ensure that when someone is admitted to hospital, the time is used effectively to avoid recurrent hospitalisation [31]. The results of the MATREX trial do not lend support to the routine use of MCP in the management of acute exacerbation of COPD this is in line with two recent systematic reviews published since the completion of this study. The pragmatic stance adopted throughout our investigation and the inclusion of both urban and rural sites with a broad range of socioeconomic characteristics means our findings are likely to have a high degree of generalisibility. It is possible that MCP may have therapeutic value to subgroups of COPD patients in specific circumstances but this has not yet been shown.

## Competing interests

The authors declare that they have no conflicts of interest. All authors have completed the Unified Competing Interest form at http://www.icmje.org/coi_disclosure.pdf (available on request from the corresponding author) and declare: no support from any organisation for the submitted work [or describe if any]; no financial relationships with any organisations that might have an interest in the submitted work in the previous three years [or describe if any], no other relationships or activities that could appear to have influenced the submitted work.

## Authors’ contributions

JC (University of East Anglia, Norwich, UK) participated in the steering committee of the MATREX trial, was responsible for writing this report, was involved in the design of the trial, and analysed data. FE (University of East Anglia, Norwich, UK) participated in the steering committee of the MATREX trial, contributed to the writing of this report, was involved in the design of the trial, and collected, verified, and analysed data. GB (University of East Anglia, Norwich, UK) participated in the steering committee of the MATREX trial, commented on a draft of this report, was involved in the design of the trial, and verified and analysed data. AC (University of East Anglia, Norwich, UK) participated in the steering committee of the MATREX trial, commented on a draft of this report, was involved in the design of the trial, and verified and analysed data. LS (University of East Anglia, Norwich, UK) participated in the steering committee of the MATREX trial, commented on a draft of this report, was involved in the design of the trial, and verified and analysed data. AB (University of East Anglia, Norwich, UK) participated in the steering committee of the MATREX trial, commented on a draft of this report, collected and verified data. MB (University of East Anglia, Norwich, UK) participated in the steering committee of the MATREX trial, commented on a draft of this report and was involved in the design of the trial. IH (University of East Anglia, Norwich, UK) participated in the steering committee of the MATREX trial, commented on a draft of this report and was involved in the design of the trial. All authors have contributed to, seen and approved the final version of the report.

## Participating centres

We have listed each hospital with the names of the local principal investigator and lead physiotherapist who supervised the enrolling of patients and adherence to the MCP treatment protocol. The figure in brackets represents the number of patients recruited into the trial. Norfolk & Norwich Hospital, Norfolk (158) S Watkin, R Ellis; James Paget Hospital, Norfolk (130) D Ellis, R Matthews; Queen Elizabeth Hospital, Norfolk (73) A Pawlowicz, J Kerrigan; University Hospital Aintree, Liverpool (166) R Angus, V Ford.

## Role of the funding source

The sponsor had no role in the analysis or in the decision to submit this paper for publication. They were involved in the original brief for the study, peer review of the protocol and final report and monitoring of the study throughout. Enrolment and data collection was undertaken by staff funded by the study grant. The corresponding author had full access to all the data in the study and had final responsibility for the decision to submit for publication.

## The MATREX trial collaboration

Chief Investigator: J Cross. MATREX Trial Co-ordinating Centre: F Elender, A Blyth, H Talbot, C Minter, K Clipsham. Trial Management Group: J Cross (Chair), I Harvey, M Bachmann, L Shepstone, A Clark, G Barton, A Blyth. Trial Steering Committee: J Cross, I Harvey, M Bachmann, L Shepstone, A Clark, G Barton, A Blyth, D Price (Chair), S Watkin, R Ellis, D Ellis, R Matthews, A Pawlowicz, J Kerrigan, R Angus, V Ford, J Close, S Olive, P Browne, K Jones. Independent Data Monitoring Committee: J Pryor (London), R Lilford (Birmingham, Chair), M Roughton (London).

## Pre-publication history

The pre-publication history for this paper can be accessed here:

http://www.biomedcentral.com/1471-2466/12/33/prepub
